# A Novel Air Quality Early-Warning System Based on Artificial Intelligence

**DOI:** 10.3390/ijerph16193505

**Published:** 2019-09-20

**Authors:** Xinyue Mo, Lei Zhang, Huan Li, Zongxi Qu

**Affiliations:** 1 College of Atmospheric Sciences, Lanzhou University, Lanzhou 730000, China; moxy16@lzu.edu.cn; 2 School of Information Science and Engineering, Lanzhou University, Lanzhou 730000, China; lih17@lzu.edu.cn; 3 School of Management, Lanzhou University, Lanzhou 730000, China; quzx14@lzu.edu.cn

**Keywords:** air pollutant concentration prediction, air quality evaluation, air pollution early-warning handbook, Jing-Jin-Ji region, smart city construction

## Abstract

The problem of air pollution is a persistent issue for mankind and becoming increasingly serious in recent years, which has drawn worldwide attention. Establishing a scientific and effective air quality early-warning system is really significant and important. Regretfully, previous research didn’t thoroughly explore not only air pollutant prediction but also air quality evaluation, and relevant research work is still scarce, especially in China. Therefore, a novel air quality early-warning system composed of prediction and evaluation was developed in this study. Firstly, the advanced data preprocessing technology Improved Complete Ensemble Empirical Mode Decomposition with Adaptive Noise (ICEEMDAN) combined with the powerful swarm intelligence algorithm Whale Optimization Algorithm (WOA) and the efficient artificial neural network Extreme Learning Machine (ELM) formed the prediction model. Then the predictive results were further analyzed by the method of fuzzy comprehensive evaluation, which offered intuitive air quality information and corresponding measures. The proposed system was tested in the Jing-Jin-Ji region of China, a representative research area in the world, and the daily concentration data of six main air pollutants in Beijing, Tianjin, and Shijiazhuang for two years were used to validate the accuracy and efficiency. The results show that the prediction model is superior to other benchmark models in pollutant concentration prediction and the evaluation model is satisfactory in air quality level reporting compared with the actual status. Therefore, the proposed system is believed to play an important role in air pollution control and smart city construction all over the world in the future.

## 1. Introduction

Air is one of the most basic elements for human survival and good air quality is necessary for human health. Unfortunately, air pollution has become a global problem, which has aroused widespread concern from scholars, governments and the public. Some studies have found that exposure to air pollutants is associated with the occurrence of many diseases such as respiratory disease, cardiovascular disease and even cancer, contributing to as many as 4–9 million human deaths per year globally [1,2]. The situation in China is also grim. With the rapid development of industrialization and urbanization, more and more fossil fuels are being burned, which results in increasing emissions of sulphur, nitrogen and particulate matter, causing deteriorating air quality and frequent hazy weather. As the “Capital Economic Circle” and future world-class urban agglomeration, influenced by adverse geographical and meteorological conditions along with industrial structure, the Jing-Jin-Ji region has become one of the most heavily polluted areas, with frequent long duration, wide range and severe degree regional pollution events. To solve this serious problem, researchers have done a lot of work, including air pollutant prediction and air quality evaluation.

Numerous forecasting models have been proposed, mainly for pollutant concentration. According to their principles, these forecasting models can be divided into three categories: statistic forecasting models, numerical forecasting models and machine learning models.

Statistic forecasting models have been widely used in air quality forecasting from the early days because of their simplicity and rapidity, and they still have value in application and research up to now. They can predict pollutant concentrations in the future only by studying the relationship between pollutant concentration and meteorological factors from past records without information about pollution sources. Common statistical models include the multiple linear regression model (MLR) [3], autoregressive integrated moving average model (ARIMA) [4], grey model (GM) and Markov model. For example, Elbayoumi et al. [5] used MLR to predict the annual indoor concentrations of PM_2.5_ and PM_10_ by analyzing the meteorological variables (wind speed, temperature and relative humidity) collected from 12 natural ventilation systems. Jian et al. [6] used ARIMA to study the effects of meteorological factors on the concentrations of ultrafine particles and PM_10_ in Hangzhou under heavy traffic conditions. A first-order variable grey differential equation model was proposed by Pai et al. [7] to predict the hourly PM concentration in Banqiao, Taiwan. A Hidden Markov Model (HMMS) was used to predict daily average PM_2.5_ concentrations [8]. Although these statistic forecasting models (linear method) have been widely used in PM concentration prediction (non-linear process), their accuracy is largely limited by their linear mapping ability. Most of the air pollutant time series in the real world are non-linear and irregular, so statistic forecasting model may not be suitable for these data.

Since the 1990s, with the development of computer technology and the abundance of air pollution data, numerical forecasting models have been greatly developed and are currently in the third generation. Based on the idea of “One Atmosphere”, they realize two-way coupling between atmospheric dynamics and atmospheric chemistry which can simulate atmospheric physical and chemical processes on different scales and therefore predict the concentrations of different air pollutants [9]. Numerical forecasting model usually consist of meteorological modules, emission modules and chemical modules following this principle that weather or climate modules provide meteorological background fields which drive the chemical transport modules. At present, common numerical forecasting models include the U.S. Models-3 and WRF-Chem, Polyphemus from France as well as Nested Air Quality Prediction Modeling System (NAQPMS) from China [10,11,12]. Although numerical forecasting models are helpful to reveal the mechanism of pollution processes, their accuracy, especially in severe air pollution incidents, is greatly limited by some difficulties such as inaccurate atmospheric boundary layer simulation schemes, insufficient emission inventory of pollution sources and limited knowledge of atmospheric physical and chemical process. Furthermore, they require a lot of computing time.

Machine learning belongs to the field of artificial intelligence. The arrival of the big data era has brought unprecedented opportunities for the development of machine learning. Machine learning has excellent performance in regression and classification problems, and it is usually recognized as one of the most powerful tools in pollutant prediction for its high robustness and fault tolerance. Therefore, there are increasingly studies on pollutant concentration prediction with machine learning models. For example, support vector machine (SVM) [13] and artificial neural network (ANN) [14] are commonly selected. Paschalidou et.al [15] used a radial basis function (RBF) and multilayer perceptron (MLP) to predict hourly concentrations of PM_2.5_ in Cyprus. Wu et al. [16] acquired predictions of PM_10_ concentrations using a general regression neural network (GRNN).

Pollutant concentration data are too abstract for the public to understand, and people are eager for simplified and intuitive information to quickly understand the state of ambient air, which means air quality evaluation is indispensable. When it comes to methods of air quality evaluation, the most commonly used method is the air quality index (AQI) originally proposed by the US Environmental Protection Agency (EPA). AQI is widely used worldwide, while the standards vary among countries. China’s standard comes from “Technical Regulation on Air Quality Index (on trial) (HJ 633-2012)” issued by the Ministry of Environmental Protection. It considers a variety of pollutants including PM_2.5_, PM_10_, NO_2_, SO_2_, CO, O_3_. However, as with all environmental quality evaluations, there are ambiguities in air quality evaluation due to the vagueness of evaluation factors, criteria and objects, etc., which makes it difficult to justify the use of sharp boundaries in classification schemes, so the air quality index method has some limitations, for example, a slight increase or decrease of pollutant data near a boundary value will change the evaluation level. Such fuzziness has led many researchers to seek advanced evaluation methods [17], for instance, fuzzy mathematics. Fuzzy mathematics is proved to be a useful tool for air quality evaluation [18,19], and many air quality indicators based on fuzziness [20,21,22,23] are proposed.

Individual prediction or evaluation is not enough to help us cope with air pollution, so an integrated and complete system is expected to play a greater value. Some early-warning systems including prediction and evaluation have been gradually proposed. The problem of air pollution in China has attracted increasing attention, but there are relatively few in-depth and targeted studies in air quality early warning based on artificial intelligence. Consideration of pollutants which affect air quality should be as comprehensive as possible, but some studies only focus on single pollutant, mainly PM. Although the selection of experimental sites is of importance, some scholars don’t give sufficient reasons such as purpose and significance for their choices. The selection of algorithms and pollutant concentration limits in air quality evaluation also remain to be discussed. Therefore, developing an accurate and robust air quality early-warning system has become an urgent need of society. It is hoped to provide not only air quality information comprehensively and objectively, but also necessary preventive measures for citizens to avoid hazards, and even help relevant departments to better control air pollution and minimize negative impacts.

Based on the above analyses, this paper proposes a novel air quality early-warning system composed of prediction and evaluation. The prediction part took advantage of advanced improved complete ensemble empirical mode decomposition with adaptive noise (ICEEMDAN) and combined whale optimization algorithm (WOA) with extreme learning machine (ELM). The three methods have been proved to be effective in air pollutant forecasting [24,25,26]. Fuzzy comprehensive evaluation (FCE) based on fuzzy mathematics was conducted subsequently.

Generally speaking, the contributions of this paper are as follows:

- A complete air quality early-warning system was established and achieved good results in the Jing-Jin-Ji region where air pollution problems are of great concern.
- A novel hybrid prediction model ICEEMDAN-WOA-ELM was proposed for the main air pollutants in Beijing, Tianjin and Shijiazhuang. ICEEMDAN and WOA are confirmed to greatly improve the prediction ability of ELM through comparison.
- The predictive results can be transformed into corresponding air quality levels by fuzzy comprehensive evaluation, which means citizens without professional knowledge of atmospheric science can easily understand the current air quality and get scientific advices to avoid air pollution.
- The air quality early-warning system is feasible and practical in air pollution treatment, which can not only protect the public from air pollution but also offer services for government decision-making on environmental protection.

The rest of this paper is organized as follows: Section 2 briefly introduces the methodologies adopted in this paper. Empirical research is given in Section 3, along with the description of experiment sites, data, evaluation criteria and so forth. Section 4 gives the conclusions.

## 2. Methodology

### 2.1. The Proposed Air Quality Early-Warning System

**Figure 1:**
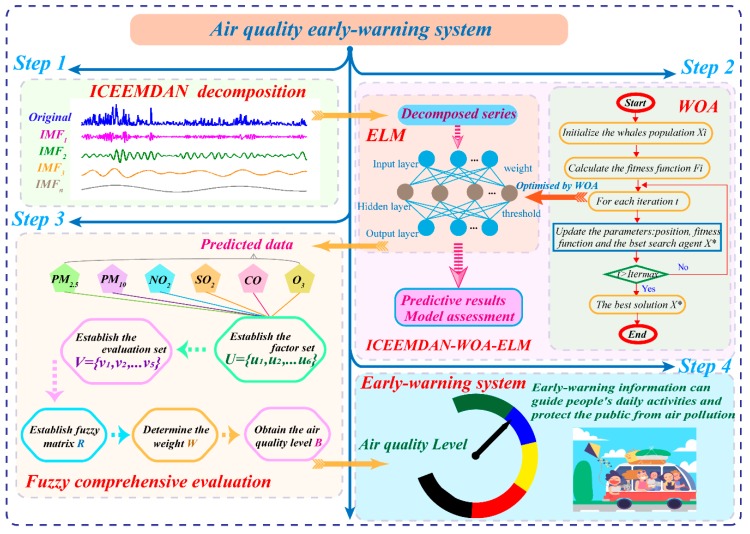
Flow diagram of the proposed system.

In this section, the air quality early-warning system whose core is the hybrid ICEEMDAN-WOA-ELM-FCE model is introduced in detail. The flow diagram consisted of four steps, presented in Figure 1.

- Step 1: Pollutant concentration data are usually chaotic time series, requiring denoising technology to eliminate the influences of outliers and improve the prediction accuracy. ICEEMDAN is used to process the original data into several IMFs from high frequency to low frequency, which contain different characteristics of the original data.
- Step 2: The ELM optimized by WOA is applied to build a predictor for each IMF. The WOA algorithm is used to obtain the best parameters of ELM to establish a forecasting model which is not only fast but also accurate. All the predictive results of IMFs are synthesized and the final predictive result is obtained. The optimized ELM model is used to forecast the concentrations of six major air pollutants in Beijing, Tianjin and Shijiazhuang, which will be the key information for the evaluation model.
- Step 3: Fuzzy comprehensive evaluation can convert the predictive results into air quality levels scientifically and objectively, providing crucial information for further research and analysis.
- Step 4: The air quality information can be applied to guide people’s daily lives. Different colors are assigned to different levels, so air quality information can be easily understood. In addition, brief but practical guidance corresponding to levels can be offered to the public against air pollution. Scientific and precise results also serve the government decision-making on environmental protection. Generally, the proposed air quality early-warning system will play a key role in future air pollution prevention.

In this section, all individual methods belonging to the air quality early-warning system are described in detail, including ICEEMDAN, WOA, ELM and FCE.

### 2.2. Improved Complete Ensemble Empirical Mode Decomposition with Adaptive Noise (ICEEMDAN)

The empirical mode decomposition (EMD) [27] is a widely used method to analyze non-linear and non-stationary data. Compared with the traditional decomposition algorithm, Fourier transform or wavelet transform which are more applicable to stationary and linear data, EMD is adaptive and highly efficient. Original data can be expressed as a sum of intrinsic mode functions (IMFs) and a final monotonic trend by EMD, but oscillations may be produced with different scales in one mode or with same scale in different modes which called “mode mixing”. The ensemble empirical mode decomposition (EEMD) [28] is proposed to address this problem by adding Gaussian white noise to the original signal, but the added noise can’t be completely neutralized and different noisy copies of the signal may produce different number of modes. The complete ensemble empirical mode decomposition with adaptive noise (CEEMDAN) [29] provides accurate reconstruction of the original signal, better spectral separation of the mode and computational efficiency, achieving huge improvements on EEMD. Furtherly, the ICEEMDAN [30] improves some aspects of CEEMDAN involving residual noise, “spurious mode” and so forth, becoming the latest decomposition method of EMD family. In this study, considering the non-stationary and non-linear characteristics of pollutant concentration, ICEEEMDAN was used as a data preprocessing method to better dig out the rules behind the pollutant data and serve the prediction later. The main steps of ICCEMDAN are summarized as follows:

(1) Calculate local means of I realizations $x^{\left(i\right)}=x+\beta_{0}E_{1}\left(w^{\left(i\right)}\right)$ by EMD to get the first residue $r_{1}=\langle M\left(x^{\left(i\right)}\right)\rangle$, where $w^{\left(i\right)}\left(i=1,\ldots ,I\right)$ is a realization of white Gaussian noise with zero mean unit variance, $E_{k}\left(.\right)$ is the operator that produces the kth mode obtained by EMD and M(.) is the operator that generates the local mean of the applied signal.

(2) Calculate the first mode $\overset{ˇ}{d_{1}}=x-r_{1}$ at the first stage (k=1).

(3) Calculate the second residue as the average of local means of the realizations $r_{1}+\beta_{1}E_{2}\left(w^{\left(i\right)}\right)$ and define the second mode: $\tilde{d_{2}}=r_{1}-r_{2}=r_{1}-\langle M\left(r_{1}+\beta_{1}E_{2}\left(w^{\left(i\right)}\right)\right)\rangle .$

(4) For k=3,…K calculate the kth residue $r_{k}=\langle M\left(r_{k-1}+\beta_{k-1}E_{k}\left(w^{\left(i\right)}\right)\right)\rangle$.

(5) Calculate the kth mode $\tilde{d_{k}}=r_{k+1}-r_{k}$.

(6) Return to step 4 for next k.

### 2.3. Whale Optimization Algorithm (WOA)

Inspired by the bubble-net hunting strategy which corresponds to the social behavior of humpback whales, a nature-inspired meta-heuristic optimization algorithm called WOA [31] was proposed in 2016. Tested with 29 mathematical benchmark functions and six structural engineering problems in exploration, exploitation, local optima avoidance and convergence behavior, WOA was proved to be highly competitive compared to the state-of-art meta-heuristic algorithms as well as conventional methods. The mathematical model of WOA is illustrated as follows [31].

#### 2.3.1. Encircling Prey

Humpback whales can identify and encircle the location of their prey. After defining the best search agent, other search agents will try to move to the best location. This behavior is expressed by the following mathematical formulas:

(1) $$\vec{D}=\left|\vec{C}\cdot \vec{X^{*}}\left(t\right)-\vec{X}\left(t\right)\right|$$

(2) $$\vec{X}\left(t+1\right)=\vec{X^{*}}\left(t\right)-\vec{A}\cdot \vec{D}$$

where t is the current iteration, $\vec{X^{*}}$ is the best position, $\vec{X}$ denotes the position vector, · is an element-by element multiplication, and $\vec{A}$ and $\vec{C}$ are coefficient vectors which can be calculated by the following equations:

(3) $$\vec{A}=2\vec{a}\cdot \vec{r}-\vec{a}$$

(4) $$\vec{C}=2\cdot \vec{r}$$

where $\vec{r}$ is a random vector between 0 and 1, and $\vec{a}$ is linearly reduced from 2 to 0 in the iteration process.

#### 2.3.2. Bubble-Net Attacking Method (Exploitation Phase)

Humpback whales usually attack their prey using the bubble-net strategy and two approaches are designed:

(1) Shrinking encircling mechanism.

This behavior is realized by reducing the value of $\vec{a}$ in Equation (3). Setting random $\vec{A}$ values in [−1,1], the new position can be obtained between the original position and the current position of the best agent.

(2) Spiral updating position

A spiral equation is established between whales and prey to simulate the helix-shaped movements of humpback whales:

(5) $$\vec{X}\left(t+1\right)=\vec{D^{\prime }}\cdot e^{bl}\cdot \cos\left(2\pi l\right)+\vec{X^{*}}\left(t\right)$$

where $\vec{D^{\prime }}=\left|\vec{X^{*}}\left(t\right)-\vec{X}\left(t\right)\right|$ is the distance between the ith whale and the best position obtained so far, b is a constant to define the logarithmic spiral, l is a random number between −1 and 1, and · is an element-by-element multiplication. WOA assumes that there is a 50% probability of choosing shrinking encircling mechanism or the spiral model to update the position of whales in the optimization process. The algorithm is defined as follows:

(6) $$\vec{X}\left(t+1\right)=\{\begin{array}{ll} \vec{X^{*}}\left(t\right)-\vec{A}\cdot \vec{D} & if\;p<0.5\\ \vec{D^{\prime }}\cdot e^{bl}\cdot \cos\left(2\pi l\right)+\vec{X^{*}}\left(t\right)\; & if\;p\geq 0.5 \end{array}$$

where p is a random number between 0 and 1.

#### 2.3.3. Search for Prey (Exploration Phase)

Humpback whales can randomly search for prey according to the position of each other. In the exploration phase, we can update the location of a search agent based on a randomly selected search agent, rather than the best search agent found so far. This mechanism emphasizes exploration, allowing the WOA algorithm to perform a global search. This mathematical model is expressed as follows:

(7) $$\vec{D}=\left|\vec{C}\cdot \vec{X_{rand}}-\vec{X}\right|$$

(8) $$\vec{X}\left(t+1\right)=\vec{X_{rand}}-\vec{A}\cdot \vec{D}$$

where $\vec{X_{rand}}$ is a random location vector selected from the current population.

The WOA algorithm (Algorithm 1) starts with a set of random solutions. In each iteration, the search agent updates its location based on the randomly selected search agent or the best solution obtained so far. A random search agent is selected when $\left|\vec{A}\right|$ > 1, and the best solution is selected when $\left|\vec{A}\right|$ < 1. According to p value, WOA can switch between spiral and circular movement. The WOA algorithm is terminated when it satisfies the termination criterion. The pseudo code of the WOA algorithm is represented as follows:

**Algorithm 1** WOA**Input:** Maximum number of iterations $Iter_{Max}$, Fitness function $F_{i}$, Current iteration number *t*,   A random number l between −1 and 1, A constant number b. 1: Initialize the whales population $X_{i}\left(i=1,2,3,\ldots ,n\right)$
2: **for** each search agent **do**3:  Calculate the fitness function $F_{i}$
4: **end for**5: $X^{*}=$ the best search agent6: **while**
$t<Iter_{Max}$
**do**7:   **for** each search agent **do**8:   Update a,A,C,l and p
9:   **if**
p<0.5
**then**10:     **if**
|A|<1
**then**11:      Update the position of search agent using Eq(2); 12:     **elseIf**
|A|≥1
13:      Select a random search agent $X_{rand}$;14:      Update position of search agent using Eq(8);15:     **end if**16:    **elseIf**
p>0.517:     Update the position with spiral Eq(5);18:    **end if**19:  **end for**20:  Check if any search agent goes beyond the search space and amend it;21:  **for** each search agent **do**22:    Calculate the fitness function $F_{i}$
23:  **end for**24:  Update $X^{*}$ if there is a better solution;25:  t=t+126: **end while**27: **return**
$X^{*}$

### 2.4. Extreme Learning Machine (ELM)

**Figure 2:**
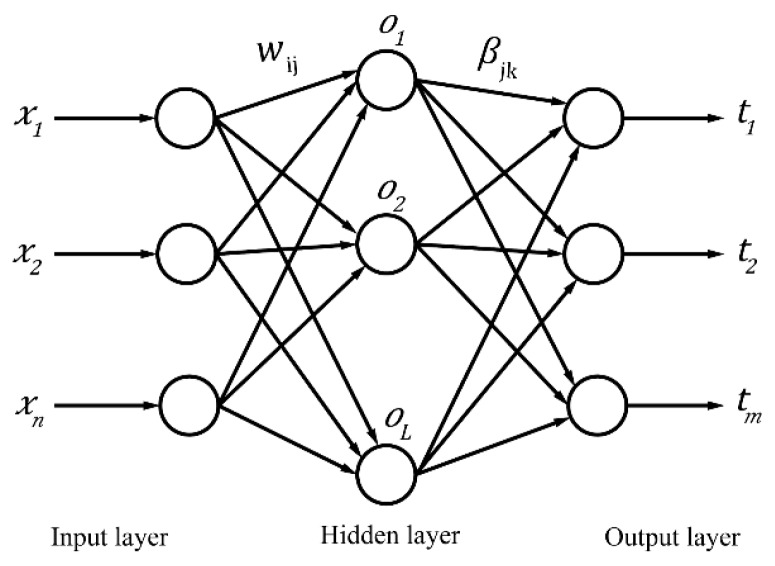
Single hidden layer feedforward neural network.

ELM [32] is a simple and extremely fast learning algorithm of single-hidden layer feedforward neural networks (SLFN). ELM randomly assign input weights and hidden layer biases (thresholds) without adjustment in the training process, which leads to thousands of times faster than traditional feedforward network learning algorithms and better generalization performance in most artificial and real benchmark problems. The structure of single-hidden layer feedforward neural network is shown in Figure 2.

For N independent samples $\left(x_{i},t_{i}\right)$, $x_{i}={\left[x_{i1},x_{i2},\ldots ,x_{in}\right]}^{T}\in R^{n}$ and $t_{i}={\left[t_{i1},t_{i2},\ldots t_{im}\right]}^{T}\in R^{m}$. SLFN can be expressed as [32]:

(9) $$\overset{L}{\underset{i=1}{\sum }}\beta_{i}g\left(w_{i}\cdot x_{j}+b_{i}\right)=o_{j},\;j=1,2,\ldots ,N$$

where $w_{i}={\left[w_{i1},w_{i2},\ldots ,w_{in}\right]}^{T}$ is the weight vector between the input layer neurons and the ith hidden layer neuron, $b_{i}$ is the threshold of the ith hidden layer neuron, g(x) is the activation function, and $\beta_{i}={\left[\beta_{i1},\beta_{i2},\ldots ,\beta_{im}\right]}^{T}$ is the weight vector between the ith hidden layer neuron and the output layer neurons. Formula (9) can be expressed as:

(10) Hβ=T

where H is the output matrix of the hidden layer, β is the weight vector between the hidden layer neurons and the output layer neurons, T is the expected output of network, represented as follows [32]:

(11) $$H={\left[\begin{array}{ccc} g\left(w_{1}\cdot x_{1}+b_{1}\right) & \cdots & g\left(w_{L}\cdot x_{1}+b_{L}\right)\\ \vdots & \cdots & \vdots \\ g\left(w_{1}\cdot x_{N}+b_{1}\right) & \cdots & g\left(w_{L}\cdot x_{N}+b_{L}\right) \end{array}\right]}_{N\times L}$$

The number of required hidden layer neurons L≤N when activation function g is infinitely differentiable. Its solution is:

(12) $$\hat{\beta }=H^{+}T$$

where $H^{+}$ is the Moore-Penrose generalized inverse of H.

ELM can generate w and b randomly before training and calculate β only by determining L and g(x). Generally, the ELM algorithm has the following steps:

(1) Determine the number of neurons L in the hidden layer, and randomly set the connection weight w between the input layer and the hidden layer and the threshold b of hidden layer neurons.

(2) An infinitely differentiable function g(x) is selected as the activation function of the hidden layer neurons, and then the output matrix H of the hidden layer is calculated.

(3) Calculate the weight of the output layer: $\hat{\beta }=H^{+}T$.

### 2.5. Fuzzy Comprehensive Evaluation (FCE)

Environmental quality is a huge and ambiguous system with a large number of uncertain factors. Fuzzy mathematics [18] can effectively solve the influences of ambiguity of evaluation boundary and monitoring error on evaluation. Using membership function to represent air quality level can eliminate subjective and artificial factors in classification, objectively reflecting regional air quality. The concrete steps of fuzzy comprehensive evaluation are as follows:

(1) Establish the factor set

A factor set is a set of elements that affect the evaluation object, usually represented by $U=\left\{u_{1},u_{2},\ldots ,u_{m}\right\}$. It is well known that different pollutants can cause different hazards to human health, so these parameters should be treated separately. Therefore, six main pollutants are selected as air quality parameters in this project:

(13) $$U=\left\{u_{1},u_{2},\ldots ,u_{6}\right\}=\left\{PM_{2.5},PM_{10},NO_{2},SO_{2},CO,O_{3}\right\}$$

(2) Set up the evaluation set

Because this research is carried out in China, air pollutant concentration limits from “Technical Regulation on Air Quality Index (on trial) (HJ 633-2012)” of China have a reference value. On account of the lack of values of O_3_ (8 h) beyond the fifth level, we have a decision that the evaluation set comprises five levels: $V=\left\{v_{1},v_{2},\ldots v_{5}\right\}=\left\{I,\;\mathrm{II},\;\mathrm{III},\;\mathrm{IV},\;V\right\}$ and the corresponding air quality categories are “Excellent, Good, Moderate, Poor, Hazardous”. The air quality levels and corresponding concentration limits of different pollutants are given in Table 1.

(1) Establish fuzzy matrix

The fuzzy matrix can be expressed by the matrix R, where $R_{ij}$ is the membership degree of factor $u_{i}$ aiming at the comment $v_{j}$:

(14) $$R={\left(r_{ij}\right)}_{m\times n}=\left[\begin{array}{cc} \begin{array}{cc} r_{11} & r_{12}\\ r_{21} & r_{22} \end{array} & \begin{array}{cc} \cdots & r_{1n}\\ \cdots & r_{2n} \end{array}\\ \begin{array}{cc} \cdots & \cdots \\ r_{m1} & r_{i2} \end{array} & \begin{array}{cc} \cdots & \cdots \\ \cdots & r_{mn} \end{array} \end{array}\right]$$

The membership function can calculate the membership degree of pollutant concentration to the evaluation grade. There are many membership functions such as halved trapezoidal distribution function, Gauss membership function, triangular membership function, etc. In this study, the halved trapezoidal distribution function [33] which has often been used in air quality evaluation is selected and details are presented as follows:

(15) $$\begin{array}{l} r_{ij}=\{(\begin{array}{c} 1\\ u_{i\left(j+1\right)}-x_{i})/\left(u_{i\left(j+1\right)}-u_{ij}\right)\\ 0 \end{array}\;\begin{array}{c} x_{i}\leq u_{ij}\\ u_{ij}<x_{i}\leq u_{i\left(j+1\right)}\\ x_{i}>u_{i\left(j+1\right)} \end{array}\;j=1\\ r_{ij}=\{\begin{array}{c} \left(x_{i}-u_{i\left(j-1\right)}\right)/\left(u_{ij}-u_{i\left(j-1\right)}\right)\\ \left(u_{i\left(j+1\right)}-x_{i}\right)/\left(u_{i\left(j+1\right)}-u_{ij}\right)\\ 0 \end{array}\;\begin{array}{c} u_{i\left(j-1\right)}\leq x_{i}\leq u_{ij}\\ u_{ij}<x_{i}\leq u_{i\left(j+1\right)}\\ x_{i}>u_{i\left(j+1\right)} \end{array}\;j=2,3,4\\ r_{ij}=\{\begin{array}{c} 0\\ \left(x_{i}-u_{i\left(j-1\right)}\right)/\left(u_{ij}-u_{i\left(j-1\right)}\right)\\ 1 \end{array}\;\begin{array}{c} x_{i}\leq u_{i\left(j-1\right)}\\ u_{i\left(j-1\right)}<x_{i}\leq u_{ij}\\ x_{i}>u_{ij} \end{array}\;j=5 \end{array}$$

(2) Determine the factor weights

The weight of a factor is an index to measure the relative degree of a pollutant impact on air quality. The multi-scale weighting method is commonly used in the fuzzy evaluation of environment quality, therefore the weight of pollution factor can be obtained by Equation (16):

(16) $$w_{i}=\left[u_{i}^{k}/\left(\frac{1}{n}\overset{n}{\underset{j=1}{\sum }}v_{ij}\right)\right]/\overset{m}{\underset{i=1}{\sum }}\left[u_{i}^{k}/\left(\frac{1}{n}\overset{n}{\underset{j=1}{\sum }}v_{ij}\right)\right]$$

(3) Evaluation result

By synthesizing the weight vector and the fuzzy matrix with the appropriate operator, the final result of the fuzzy comprehensive evaluation can be obtained. The Zadeh operator M(∧,∨) is commonly used as a solution, therefore it is adopted here:

(17) $$B=W⊕R=\left(w_{1},w_{2},\ldots w_{m}\right)\;\left(\wedge ,\vee \right)\;\left[\begin{array}{cc} \begin{array}{cc} r_{11} & r_{12}\\ r_{21} & r_{22} \end{array} & \begin{array}{cc} \cdots & r_{1n}\\ \cdots & r_{2n} \end{array}\\ \begin{array}{cc} \cdots & \cdots \\ r_{m1} & r_{m2} \end{array} & \begin{array}{cc} \cdots & \cdots \\ \cdots & r_{mn} \end{array} \end{array}\right]$$

According to the principle of maximum membership degree, the maximum value of B is the result of fuzzy comprehensive evaluation of air quality.

## 3. Experimental Results and Analysis

**Figure 3:**
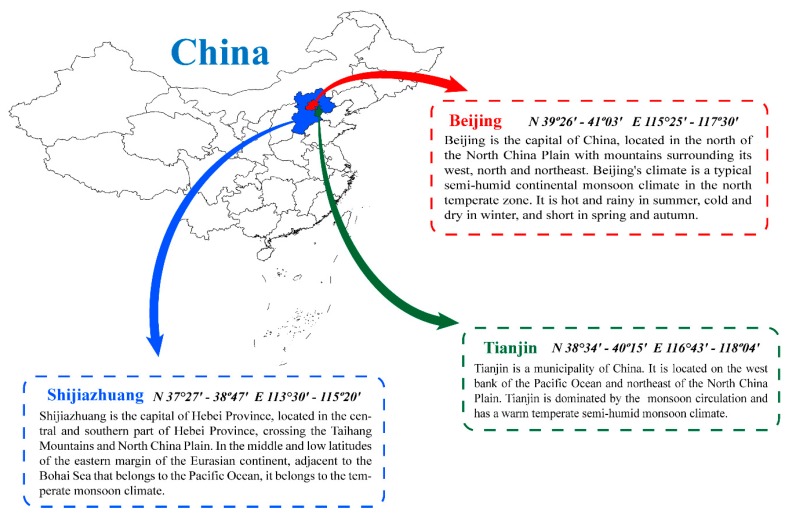
Locations and climatic conditions of the study areas.

In this section, in order to evaluate the performance of proposed air quality early-warning system, three datasets from three cities (Beijing, Tianjin, Shijiazhuang) in China were used in case studies (the simple map of the study areas is displayed in Figure 3. The main reasons for the choice are: (1) Jing-Jin-Ji region is a Beijing-centered world-class urban agglomeration which has a developed economy and important strategic position. It covers 13 cities, 110 million people and 218,000 km^2^ of land area, so air pollution is really of concern here. (2) In this region, the heavy industrial structure, dense population and limited environmental capacity have led to frequent haze events which cause serious troubles to people’s normal life and social development. At the moment, how to balance economic development and environmental protection is urgent and it is hoped our system will be beneficial for air pollution control. (3) Influenced by meteorological conditions, pollutant emissions and transport, secondary transformation of particulate matter, synthetical effect of nature and human, the air pollution is extremely complex and prominent here. This problem not only seriously endangers human health and economic development, but also has impacts on climate and environment change. Therefore, relevant research conducted in this region is representative and referential for air pollution control of other metropolis in the world.

### 3.1. Dataset Description

Datasets used in this study were from the Ministry of Ecology Environment of China including daily concentration of six main air pollutants in three cities from 1 September 2016 to 30 September 2018. For missing data, the nearby mean was used as the missing data. The sample size of one pollutant in one city was 760, which were divided into training set (699) from 1 September 2016 to 31 July 2018 and testing set (61) from 1 August 2018 to 30 September 2018. The characteristics of the whole samples including maximum (Max), minimum (Min), mean (Mean) and standard deviation (Std.) are shown in Table 2.

### 3.2. Evaluation Criteria

To evaluate the performance of the proposed system in forecast, a set of four criteria [34] are applied: Mean absolute error (MAE), Root mean square error (RMSE), Mean absolute percentage error (MAPE) and Theil’s inequality coefficient (TIC). MAE reflects the difference between the predicted and actual value. RMSE reflects the extent of the difference between the predicted and actual values. MAPE is an index to measure the forecasting accuracy of a model in statistics. TIC is an indicator used to measure the predictive capability of a model. For all criteria, the smaller the value is, the better predictive performance the model has.

- Mean absolute error (MAE):
  (18) $$MAE=\frac{1}{N}\overset{N}{\underset{i=1}{\sum }}\left|\hat{F_{i}}-F_{i}\right|$$
- Root mean square error (RMSE):
  (19) $$RMSE=\sqrt{\frac{1}{N}\overset{N}{\underset{i=1}{\sum }}{\left(\hat{F_{i}}-F_{i}\right)}^{2}}$$
- Mean absolute percentage error (MAPE):
  (20) $$MAPE=\frac{1}{N}\overset{N}{\underset{I=1}{\sum }}\left|\frac{\hat{F_{i}}-F_{i}}{F_{i}}\right|$$
- Theil’s inequality coefficient (TIC):
  (21) $$TIC=\frac{\sqrt{\frac{1}{N}\sum_{i=1}^{N}{\left(\hat{F_{i}}-F_{i}\right)}^{2}}}{\sqrt{\frac{1}{N}\sum_{i=1}^{N}{\hat{F_{i}}}^{2}}+\sqrt{\frac{1}{N}\sum_{i=1}^{N}F_{i}{}^{2}}}$$
  where N is the number of data, $\hat{F_{i}}$ and $F_{i}$ are the predicted and actual value at time i, respectively.

### 3.3. Diebold-Mariano (D-M) Test

The Diebold-Mariano test [35] is a hypothesis test that is employed to evaluate the significance of the performance of proposed model compared with other models. The hypothesis test is defined as follows:

(22) $$H_{0}:E\left[l\left(\varepsilon_{n+t}^{1}\right)\right]=E\left[l\left(\varepsilon_{n+t}^{2}\right)\right]$$

(23) $$H_{1}:E\left[l\left(\varepsilon_{n+t}^{1}\right)\right]\neq E\left[l\left(\varepsilon_{n+t}^{2}\right)\right]$$

where l is the loss function, $\varepsilon_{n+t}^{1}$ and $\varepsilon_{n+t}^{2}$ are the forecast errors of two forecasting models. Each forecast accuracy is evaluated by an appropriate loss function, and the commonly used loss function is the MAE (Equation (18)) function [36]. For given significance level, the null hypothesis indicates that there is no significant difference between proposed model and comparison model in predictive performance.

The statistical function of the DM test is as follows:

(24) $$DM=\frac{\sum_{t=1}^{T}\left(l\left(\varepsilon_{n+t}^{1}\right)-l\left(\varepsilon_{n+t}^{2}\right)\right)/T}{\sqrt{s^{2}/T}}s^{2}$$

where $s^{2}$ is the estimate of variance of $D_{i}=l\left(\varepsilon_{n+t}^{1}\right)-l\left(\varepsilon_{n+t}^{2}\right)$. The null hypothesis is that the two prediction models have the same predictive accuracy. The DM statistic converges to the standard normal distribution N(0,1), and the null hypothesis will be rejected if $\left|DM\right|>Z_{\alpha /2}$. $Z_{\alpha /2}$ denotes the critical z-value of the standard normal distribution, and α is the significance level.

### 3.4. Case Studies

In this paper, case studies were carried out to measure the performance of forecasting model. Single model and hybrid model including ARIMA [37], GRNN [38], ELM [26], GA-ELM, WOA-ELM and EEMD-WOA-ELM were used as benchmarks to assess the proposed hybrid model. The experiment was first conducted in Beijing to verify the predictive performance of the model in details, and then experiments in Tianjin and Shijiazhuang were used to prove universality. If the proposed model outperforms other models in all case studies, we can certainly draw the conclusion that the proposed model has not only high accuracy but also universal applicability in different environments. Meanwhile, the model was assessed by the statistical test based on DM test. Furthermore, the trial-and-error method was used to determine the best experimental parameters which are listed in Table 3.

#### 3.4.1. Case Study One: Beijing

**Figure 4:**
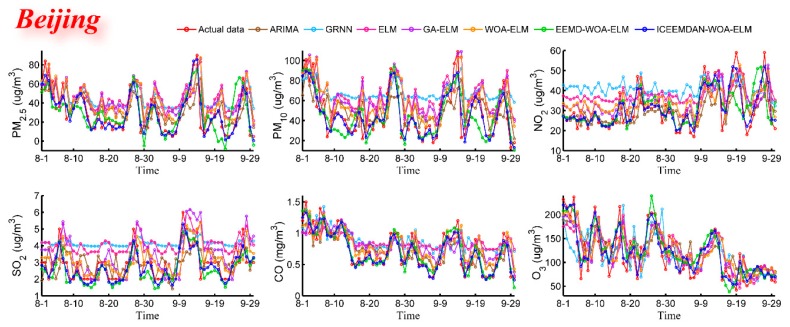
Predictive results (Beijing).

**Figure 5:**
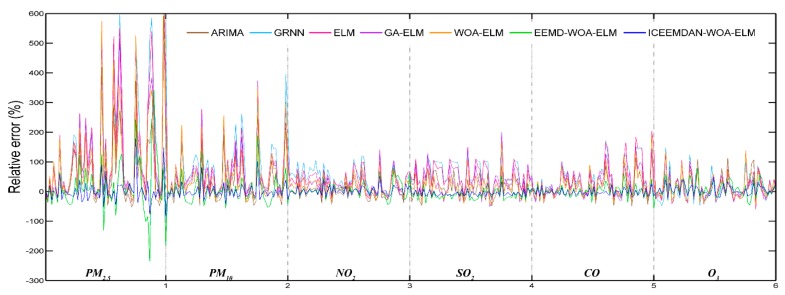
Daily relative error (Beijing).

The daily concentrations of six air pollutants from 1 September 2016 to 31 September 2018 in Beijing were employed to verify the forecasting performance of the proposed hybrid model. Daily pollutant concentrations of two months from 1 August 2018 to 31 September 2018 were predicted and compared with actual data. Figure 4 shows the predictive results and Figure 5 shows the daily relative errors (relative error = (predicted value − actual value)/actual value). In addition, four performance indicators are calculated and given in Table 4. At the same time, Table 4 also shows the predictive effectiveness of ARIMA, GRNN, ELM, GA-ELM, WOA-ELM, EEMD-WOA-ELM and ICCEMDAN-WOA-ELM as comparison, and the bold values represent the best values for each criterion. It is evident that ICEEMDAN-WOA-ELM model has the most excellent performance among all models. Its predictive results are very closer to actual values than other models. Influenced by many factors, though relative errors of PM_2.5_ and PM_10_ are larger than that of other pollutants for the highly nonlinear and non-stationary characteristics, the proposed model is more satisfactory.

Based on the information in Figure 4, Figure 5 and Table 4, it is clear that the proposed model obtains the best results for all evaluation indicators. Therefore, we can conclude that the proposed ICEEMDAN-WOA-ELM model is superior to benchmark models in the prediction of air pollutant concentrations. More comparative analyses are presented as follows:

(1) As one of time series forecasting models, ARIMA is superior to single artificial intelligence models in accuracy. The four indexes (MAE, RMSE, MAPE, TIC) of ARIMA are almost better than those of single artificial intelligence models, which is attributed to the high volatility and irregularity of air pollutant concentration data. The results show that single artificial intelligence models cannot meet the requirements of air pollutant prediction which means it is urgent to develop a hybrid model to improve the predictive performance.

(2) From the comparison between ELM and GA-ELM as well as WOA-ELM, we can conclude that optimization algorithms can really help neural network model improve performance. The ELM optimized by GA or WOA provides better predictive results for six air pollutants. For example, in PM_2.5_ forecast, the MAE, RMSE, MAPE, TIC are 17.0523, 21.7668, 125.3109, 0.2562 and 16.1554, 21.1993, 116.7680, 0.2531 for GA-ELM and WOA-ELM respectively, with moderate improvements compared with 19.1736, 23.3620, 143.0254, 0.2694 of ELM. From the comparative indicators, we can also see that WOA has better optimization capability as it can avoid local optima and maintain fast convergence. Compared with other optimization algorithms, WOA not only is simple, flexible and effective, but also can achieve a balance between exploration and exploitation.

(3) It can be clearly seen that the data preprocessing algorithm has brought a great improvement to the neural network model. Compared with other models, EEMD-WOA-ELM and ICEEMDAN-WOA-ELM are so outstanding in prediction, which fully proves the concept “decomposition and integration” or “divide and conquer” to be effective for establishing a robust air pollutant prediction model. It is obvious that model with ICEEMDAN performs better than the counterpart with EEMD in any cases. For instance, four metrics are 14.1866, 17.2348, 13.9759, 0.0665 and 21.1571, 24.5963, 21.8809, 0.0938, respectively, for O_3_. The results show that the decomposition method can greatly reduce predictive errors of model, and moreover, ICEEMDAN is superior to other decomposition methods in data decomposition.

Through the above analyses, MAE, RMSE, MAPE and TIC were used to prove the proposed ICEEMDAN-WOA-ELM hybrid model is obviously superior to all considered benchmark models for its higher accuracy and stability. Compared with single model, all models based on “decomposition and integration” framework have better predictive effectiveness, which shows this framework can effectively improve the model performance. The proposed forecasting model fits all the data of air pollutants with high volatility and irregularity, so it is qualified as the prediction part of air quality early-warning system.

#### 3.4.2. Case Study Two: Tianjin and Shijiazhuang

**Figure 6:**
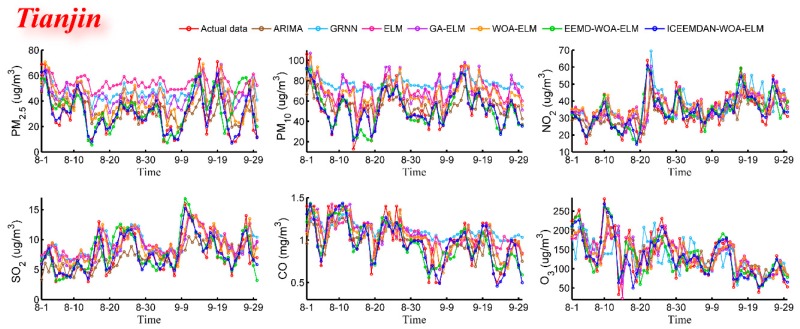
Predictive effectiveness (Tianjin).

**Figure 7:**
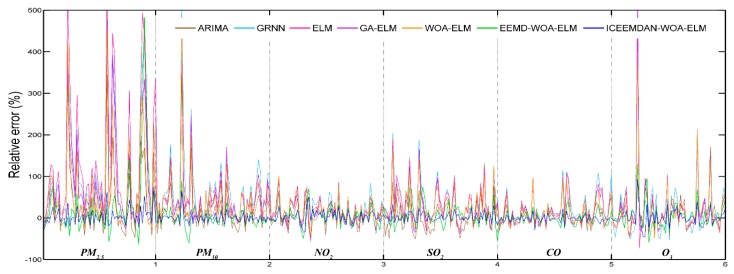
Daily relative error (Tianjin).

**Figure 8:**
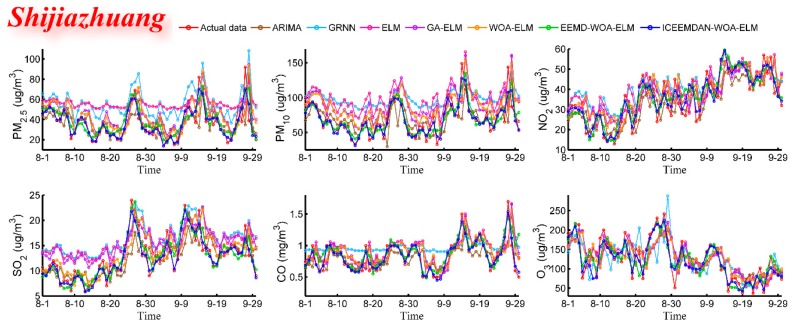
Predictive results (Shijiazhuang).

**Figure 9:**
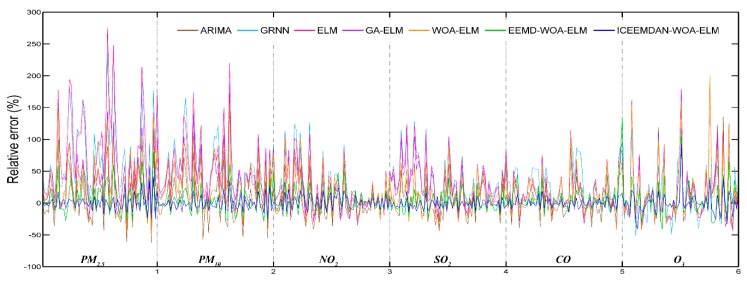
Daily relative error (Shijiazhuang).

In order to verify the predictive and universal capabilities of the proposed model, the daily air pollutant concentration data of Tianjin and Shijiazhuang (from 1 September 2016 to 30 September 2018) were also used in case studies. The main purpose is to test the generality of model under different environments. The predictive results are shown in Figure 6, Figure 7, Figure 8 and Figure 9 and Table 5 and Table 6. In Table 5 and Table 6, bold values represent the best values for each criterion among all models. From predictive results, we can see at a glance that the proposed model has better predictive results and predicted values are much closer to real values.

These experiments lead to the same conclusion as case study one that the hybrid model ICEEMDAN-WOA-ELM is superior to all listed benchmark models. For example, for the PM_10_ forecast in Tianjin and Shijiazhuang, four metric values (MAE, RMSE, MAPE, TIC) of the proposed hybrid model are 3.9662, 5.3299, 7.8186, 0.0471 and 6.3997, 8.9303, 8.9955, 0.0613, respectively, which are much lower than that of other models, and the same is true for other pollutants. Overall, different denoising methods and optimization algorithms lead to the large gaps in predictive performance. Obviously, the model has great accuracy, stability, applicability and can be well adapted to various pollutants under different environments.

Four typical evaluation indicators (MAE, RMSE, MAPE and TIC) were used to measure the performance of all models. The hybrid model ICEEMDAN-WOA-ELM performs best for it has the greatest evaluation criteria. Through these experiments, we can reasonably draw following conclusions: data preprocessing algorithm and optimization algorithm can significantly improve the predictive performance of the model. The proposed model with excellent predictive performance will be the bedrock of establishing air quality early-warning system. In addition, for their universality, these methods can be combined with some basic models to meet the needs of other research fields.

#### 3.4.3. Diebold-Mariano Test

In this section, Diebold-Mariano test was used to examine the effectiveness of the proposed hybrid model. DM test is employed to test under which circumstance an experiment will enable us to reject null hypothesis at a given significance level. The detailed description of DM test is presented in Section 3.3. The null hypothesis (Equation (22)) here is that there is no significant difference between the two models. Table 7 shows the DM test statistic value based on the MAE (Equation (18)) function. The DM values from all models are greater than the upper limits at the 1% significance level, which reflects that the proposed hybrid model significantly outperforms other comparison models.

### 3.5. Fuzzy Comprehensive Evaluation of Air Quality

In this section, predicted data of September 2018 were used for fuzzy comprehensive evaluation and further analysis. This work could visualize the predicted results of three cities. Limited by the length of paper, we had to take only the results of twenty days in September 2018 as examples which included evaluation results based on predicted and actual value as comparison to get the accuracy of the model in level forecast. Firstly, according to the methodology of fuzzy comprehensive evaluation described in Section 2.4, the evaluation set V = {I, II, III, IV, V} was established. Secondly, the membership degree of each factor to each evaluation level was calculated by the membership degree formula, and the fuzzy matrix R was established. Thirdly, the weight of pollution factor value calculated by multi-scale weighting method was an index to measure the relative degree of environmental hazards which greatly affected the evaluation result. Finally, according to the fuzzy matrix and weight index, the membership degree of evaluation level and air quality level were obtained. Evaluation results of Beijing are shown in Table 8. Taking the result of one day (01/09/2018) as an example, the probability of air quality as “I” is 0.3759, and the probability of “II”, “III”, “IV” and “V” are 0.3409, 0, 0 and 0 respectively. According to the principle of maximum membership degree, the comprehensive evaluation level of air quality should be “I” and the corresponding category is “Excellent”.

The consistency ratio of the two results in Beijing is 27/30 (90%), which not only shows the high accuracy of the proposed hybrid model in level forecast but also indirectly proves predicted concentration data are so accurate that they can fully satisfy the need of air quality early warning. Using the same algorithm, the fuzzy comprehensive evaluation of air quality for Tianjin and Shijiazhuang were conducted and the results are shown in Table 9 and Table 10. Overall, the evaluation results are basically same in these two cities. The consistency rates in Tianjin and Shijiazhuang are 26/30 (87%) and 30/30 (100%), respectively.

Therefore, the evaluation method can effectively link the pollutant concentration prediction with air quality early warning. Nevertheless, precise predictions of pollutant concentration and air quality level are not enough, because achievements of scientific research are expected to truly serve the society. Further work can be performed based on former research which means more intuitive air quality information can be released and public alarms can be issued. Therefore, an air pollution early-warning handbook was compiled and details are shown in Table 11. This work can not only guide people’s daily activities against air pollution but also provide decision-making support for government such as evaluate whether the air quality of a city meets the criteria or which temporary but mandatory measures should be taken to address potential air pollution problems.

Tips: six major air pollutants include PM_2.5_, PM_10_, NO_2_, SO_2_, CO and O_3_. It is necessary to know their characteristics:

- PM_2.5_: namely fine particulate matter, particle size less than or equal to 2.5 μm, it has smaller size, larger area, stronger activity, easier to attach toxic and harmful substances, longer residence time and transportation distance in atmosphere which mean more harmful to human health and air quality than PM_10_, can enter bronchioles and alveoli causing cardiopulmonary disease and even lung cancer.
- PM_10_: namely inhalable particulate matter, particle size less than or equal to 10 μm, can reduce the atmospheric visibility, enter upper respiratory tract causing respiratory disease.
- NO_2_: rufous and irritating odor, can promote acid rain and ozone, damage respiratory tract.
- SO_2_: colorless and irritating odor, can be oxidized into sulfuric acid mist (acid rain) or sulfate aerosol, cause respiratory diseases and cancer.
- CO: colorless and tasteless, mainly from uncompleted combustion, cause suffocation even death.
- O_3_: light blue with special odor, major constituent of photochemical smog, damage human mucosa and respiratory tract.

## 4. Conclusions

Air pollution is a long-standing problem that plagues the whole world, seriously harming human health, social development and natural environment. In order to solve this problem, a great deal of manpower and material resources have been invested, but unfortunately the results are not satisfactory enough. There is always a way and the rapid development of artificial intelligence in recent years has brought new hope for air pollution control. This proposed air quality early-warning system is hoped to play a key role in future for its accuracy and effectiveness. This system mainly consists of two parts: prediction model and evaluation model.

In order to establish the prediction model, ELM, which is famous for accuracy and robustness, was employed. Taking ELM as the core, a hybrid model ICEEMDAN-WOA-ELM was proposed. Firstly, according to the theory of “decomposition and integration”, the original time series of pollutant concentration were decomposed into IMFs by decomposition algorithm (ICEEMDAN). Secondly, the ELM optimized by WOA was used to predict each IMF. Finally, all the predictive results were combined to get the final predictive result. In this study, six main air pollutants PM_2.5_, PM_10_, NO_2_, SO_2_, CO and O_3_ in Beijing, Tianjin and Shijiazhuang were chosen. This proposed prediction model was used to predict air pollutant concentrations and compare with the six benchmark models including ARMA, GRNN, ELM, GA-ELM, WOA-ELM and EEMD-WOA-ELM. The simulation results showed that the proposed ICEEMDAN-WOA-ELM model was superior to other models and ICEEMDAN decomposition algorithm along with WOA optimization algorithm played important roles in improving the prediction accuracy of neural network.

In addition to prediction of air pollutant concentration, air quality evaluation was an indispensable part of the air early warning system. For the sake of understanding the future state of air, air quality was evaluated with the above predicted data by fuzzy comprehensive evaluation. The evaluation results were satisfactory enough compared with the actual status, which means our proposed evaluation model can meet the requirement of early warning. Furthermore, air pollution early-warning handbook was compiled to provide the public with intuitive air quality information and reasonable measures.

The combination of air pollutant prediction and air quality evaluation lays a solid foundation for the establishment and implementation of air quality early-warning system. The proposed system can offer us accurate air pollutant concentration prediction, correct air quality evaluation, reasonable countermeasures and scientific decision-making support, which means it will become a sharp weapon for air pollution control and even smart city construction in future.

## Figures and Tables

**Table 1:** The air quality level and corresponding concentration limit (units: μg/m^3^, CO (mg/m^3^)).

Level	Category	PM_2.5_	PM_10_	NO_2_	NO_2_	NO_2_	O_3_
I	Excellent	35	50	40	50	2	100
II	Good	75	150	80	150	4	160
III	Moderate	115	250	180	475	14	215
IV	Poor	150	350	280	800	24	265
V	Hazardous	250	420	565	1600	36	800

**Table 2:** The statistical properties of air pollutant concentration.

City	Pollutant Concentration ((μg/m^3^), CO (mg/m^3^))						
	Indicator	PM_2.5_	PM_10_	NO_2_	SO_2_	CO	O_3_
Beijing	Max	454	840	155	84	8	311
	Min	5	7	7	2	0.2	3
	Mean	61.2	89.8	45.5	7.2	1.0	98.4
	Std.	57.5	72.8	22.2	7.2	0.8	63.3
Tianjin	Max	290	931	132	89	9	282
	Min	8	11	14	2	0.3	3
	Mean	62.3	97.3	48.6	15.3	1.3	105.6
	Std.	47.8	68.3	21.6	11.5	0.8	61.5
Shijiazhuang	Max	621	870	183	153	10	297
	Min	12	22	13	5	0.3	6
	Mean	91.5	160.8	53.1	31.4	1.5	106.1
	Std.	82.0	118.0	24.2	24.1	1.1	68.2

**Table 3:** Experimental parameters.

Parameter	PM_2.5_	PM_10_	NO_2_	SO_2_	CO	O_3_
Input variable	4	4	8	3	8	3
Number of search agents	10	10	10	10	10	10
MaxIter of WOA	200	200	200	200	200	200
MaxIter of ICEEMDAN	1000	1000	1000	1000	1000	1000

MaxIter: maximum iteration; WOA: Whale Optimization Algorithm; ICEEMDAN: Improved Complete Ensemble Empirical Mode Decomposition with Adaptive Noise.

**Table 4:** Predictive effectiveness (Beijing).

Pollutant	Criterion	ARIMA	GRNN	ELM	GA-ELM	WOA-ELM	EEMD-WOA-ELM	ICEEMDAN-WOA-ELM
PM_2.5_	MAE	14.6003	19.5838	19.1736	17.0523	16.1554	11.2074	**5.9228**
	RMSE	18.5605	23.7382	23.3620	21.7668	21.1993	15.0801	**7.7563**
	MAPE	89.4611	155.6999	143.0254	125.3109	116.7680	60.3403	**28.8783**
	TIC	0.2445	0.2772	0.2694	0.2562	0.2531	0.2019	**0.1038**
PM_10_	MAE	17.0591	25.0788	20.8773	19.3449	18.0783	11.7358	**6.8349**
	RMSE	21.4795	29.0880	26.4483	25.2979	23.6904	14.4054	**8.6609**
	MAPE	47.6182	83.1894	67.8279	63.4524	57.3004	30.2585	**16.8055**
	TIC	0.2011	0.2364	0.2169	0.2107	0.2032	0.1375	**0.0830**
NO_2_	MAE	0.7594	1.4453	1.3793	1.0707	0.7643	0.4734	**0.2982**
	RMSE	0.9677	1.5697	1.5207	1.3447	1.0222	0.5826	**0.3717**
	MAPE	27.7888	60.4805	56.8319	39.4689	28.0332	16.1434	**9.9484**
	TIC	0.1580	0.2164	0.2098	0.1998	0.1617	0.1006	**0.0631**
SO_2_	MAE	6.6864	11.8116	9.9558	7.6664	7.3561	5.6606	**3.2131**
	RMSE	8.6302	13.4116	11.3911	9.5799	8.9238	7.8410	**4.1274**
	MAPE	22.1293	46.2987	38.7423	28.5756	27.1341	18.4094	**10.7605**
	TIC	0.1362	0.1818	0.1596	0.1405	0.1315	0.1203	**0.0632**
CO	MAE	0.1856	0.2648	0.2465	0.2105	0.1952	0.0972	**0.0740**
	RMSE	0.2390	0.2990	0.2916	0.2476	0.2404	0.1167	**0.0915**
	MAPE	30.894	50.8398	50.3263	37.5665	34.6805	15.3490	**10.3210**
	TIC	0.1499	0.1770	0.1742	0.1509	0.1474	0.0737	**0.0586**
O_3_	MAE	0.1856	0.2648	0.2465	0.2105	0.1952	0.0972	**0.0740**
	RMSE	0.2390	0.2990	0.2916	0.2476	0.2404	0.1167	**0.0915**
	MAPE	30.894	50.8398	50.3263	37.5665	34.6805	15.3490	**10.3210**
	TIC	0.1499	0.1770	0.1742	0.1509	0.1474	0.0737	**0.0586**

ARIMA: Autoregressive Integrated Moving Average; GRNN: Generalized Regression Neural Network; ELM: Extreme Learning Machine; GA: Genetic Algorithm; WOA: Whale Optimization Algorithm; EEMD: Ensemble Empirical Mode Decomposition; ICEEMDAN: improved complete ensemble empirical mode decomposition with adaptive noise; MAE: Mean absolute error; RMSE: Root mean square error; MAPE: Mean absolute percentage error; TIC: Theil’s inequality coefficient. Bold values represent the best values for each criterion among all models.

**Table 5:** Predictive effectiveness (Tianjin).

Pollutant	Criterion	ARIMA	GRNN	ELM	GA-ELM	WOA-ELM	EEMD-WOA-ELM	ICCEMDAN-WOA-ELM
PM_2.5_	MAE	11.1395	23.9600	18.3412	15.6653	13.1864	9.4036	**3.6645**
	RMSE	14.0459	26.7422	21.0818	18.2338	16.4877	12.6058	**4.6569**
	MAPE	54.9751	136.5288	106.1613	88.4899	72.3501	46.5237	**14.3900**
	TIC	0.2005	0.2982	0.2546	0.2276	0.2131	0.1743	**0.0669**
PM_10_	MAE	12.7673	24.9898	20.4742	17.8837	16.3696	7.4594	**3.9662**
	RMSE	15.9043	28.5802	24.1671	22.3880	20.5478	9.5307	**5.3299**
	MAPE	30.3552	65.0635	52.6416	45.1890	42.6398	14.6617	**7.8186**
	TIC	0.1388	0.2106	0.1837	0.1733	0.1624	0.0844	**0.0471**
NO_2_	MAE	2.1836	2.5998	2.5202	2.3106	2.1731	1.2511	**0.6417**
	RMSE	2.7434	3.1492	3.0093	2.8300	2.0674	1.6499	**0.7914**
	MAPE	30.1119	44.1561	42.9450	37.7425	35.5006	18.3743	**9.2402**
	TIC	0.1736	0.1765	0.1681	0.1625	0.1528	0.0979	**0.0478**
SO_2_	MAE	6.3905	8.5673	7.8709	7.8024	7.5693	5.0008	**3.4196**
	RMSE	8.6427	10.9683	9.7383	9.8299	9.3928	6.3693	**4.3845**
	MAPE	19.4000	27.1388	25.8628	25.2229	25.2437	15.8650	**10.9658**
	TIC	0.1268	0.1540	0.1340	0.1364	0.1289	0.0894	**0.0619**
CO	MAE	0.1690	0.2124	0.1862	0.1830	0.1832	0.1071	**0.0613**
	RMSE	0.2127	0.2645	0.2321	0.2287	0.2244	0.1256	**0.0834**
	MAPE	19.5594	27.9640	23.8661	22.6987	22.4912	12.0626	**6.4375**
	TIC	0.1051	0.1235	0.1105	0.1090	0.1078	0.0624	**0.0413**
O_3_	MAE	36.9855	45.531	37.6976	36.3093	33.8067	23.4066	**16.9133**
	RMSE	46.9635	56.3589	48.6523	48.3885	43.7632	29.5649	**22.2439**
	MAPE	39.8767	42.4992	39.7669	38.3299	34.7636	21.4147	**14.7261**
	TIC	0.1606	0.1999	0.1688	0.1689	0.1531	0.1029	**0.0779**

Bold values represent the best values for each criterion among all models.

**Table 6:** Predictive effectiveness (Shijiazhuang).

Pollutant	Criterion	ARIMA	GRNN	ELM	GA-ELM	WOA-ELM	EEMD-WOA-LM	ICCEMDAN-WOA-ELM
PM_2.5_	MAE	10.4859	21.7164	21.4921	12.7615	12.8217	6.7221	**3.6732**
	RMSE	15.3272	24.7551	24.6503	16.9633	16.8340	8.8484	**5.5817**
	MAPE	30.3538	80.5130	74.7687	41.2516	39.6367	20.4192	**9.6500**
	TIC	0.1944	0.2502	0.2514	0.1952	0.1956	0.1096	**0.0708**
PM_10_	MAE	16.0775	32.3695	31.6741	23.5597	22.3872	10.0835	**6.3997**
	RMSE	23.4520	35.6565	35.8119	29.3459	27.5434	13.7531	**8.9303**
	MAPE	25.0404	57.2783	54.0533	39.1774	37.2602	14.7342	**8.9955**
	TIC	0.1601	0.2050	0.2053	0.1772	0.1689	0.0939	**0.0613**
NO_2_	MAE	2.5505	4.2360	3.7139	3.6167	2.5586	1.4341	**0.8704**
	RMSE	3.3275	4.7459	4.3104	4.2334	3.3493	1.7469	**1.0678**
	MAPE	20.2175	40.3479	35.7381	34.7040	21.7254	11.6619	**6.8435**
	TIC	0.1263	0.1575	0.1464	0.1445	0.1212	0.0647	**0.0402**
SO_2_	MAE	7.0109	8.4042	8.0516	6.9242	6.6580	4.2502	**2.9950**
	RMSE	8.5705	10.1475	9.6071	8.5093	8.3986	5.0193	**3.3823**
	MAPE	21.1643	31.5203	28.5835	24.0445	23.3857	13.7525	**9.2431**
	TIC	0.1170	0.1279	0.1227	0.1111	0.1087	0.0662	**0.0448**
CO	MAE	0.1537	0.2320	0.1770	0.1716	0.1633	0.0992	**0.0469**
	RMSE	0.2132	0.2621	0.2340	0.2384	0.2231	0.1477	**0.0606**
	MAPE	18.9156	30.0013	23.5163	22.4597	21.4932	13.6973	**5.8451**
	TIC	0.1255	0.1421	0.1289	0.1330	0.1251	0.0836	**0.0351**
O_3_	MAE	33.2598	36.6192	34.486	33.1336	32.2717	18.0171	**13.3396**
	RMSE	41.6933	44.6073	42.289	41.2401	40.1199	22.4609	**17.9202**
	MAPE	36.7295	35.1486	36.6671	35.9133	34.2240	18.2649	**12.8219**
	TIC	0.1531	0.1663	0.1570	0.1508	0.1485	0.0842	**0.0675**

Bold values represent the best values for each criterion among all models.

**Table 7:** Diebold-Mariano test of seven models.

Model	DM Value
ARIMA	4.490165 *
GRNN	6.575409 *
ELM	6.162329 *
GA-ELM	5.093331 *
WOA-ELM	4.742791 *
EEMD-WOA-ELM	3.587006 *
ICEEMDAN-WOA-ELM	-

* Denotes the 1% significance level.

**Table 8:** Air quality evaluation results of Beijing.

Date	Predicted Value						Actual Value					
	I	II	III	IV	V	Level	I	II	III	IV	V	Level
2018/9/1	0.3759	0.3409	0	0	0	I	0.2879	0.3823	0	0	0	II
2018/9/2	0.3420	0.0630	0	0	0	I	0.3805	0.2167	0	0	0	I
2018/9/3	0.3996	0	0	0	0	I	0.3530	0	0	0	0	I
2018/9/4	0.5586	0.1814	0	0	0	I	0.5055	0	0	0	0	I
2018/9/5	0.5250	0.1205	0	0	0	I	0.4839	0.2333	0	0	0	I
2018/9/6	0.4711	0	0	0	0	I	0.4105	0	0	0	0	I
2018/9/7	0.5059	0	0	0	0	I	0.5125	0	0	0	0	I
2018/9/8	0.4855	0	0	0	0	I	0.4675	0	0	0	0	I
2018/9/9	0.4009	0.4009	0	0	0	I	0.3962	0.3962	0	0	0	I
2018/9/10	0.3367	0.3367	0	0	0	I	0.3067	0.3067	0	0	0	I
2018/9/11	0.3363	0.3363	0	0	0	I	0.3377	0.3377	0	0	0	I
2018/9/12	0.3094	0.3398	0.0035	0	0	II	0.2744	0.3885	0.0727	0	0	II
2018/9/13	0.1892	0.3649	0.2231	0	0	II	0.2123	0.3418	0.1818	0	0	II
2018/9/14	0.2579	0.3874	0.2620	0	0	II	0.2174	0.3750	0.3894	0	0	III
2018/9/15	0.3459	0.1011	0	0	0	I	0.3840	0	0	0	0	I
2018/9/16	0.4600	0	0	0	0	I	0.4532	0	0	0	0	I
2018/9/17	0.3094	0.0540	0	0	0	I	0.3267	0.0250	0	0	0	I
2018/9/18	0.2673	0.2636	0	0	0	I	0.2722	0.2500	0	0	0	I
2018/9/19	0.2867	0.2427	0	0	0	I	0.2755	0.2749	0	0	0	I
2018/9/20	0.3129	0.0907	0	0	0	I	0.2954	0.2954	0	0	0	I

**Table 9:** Air quality evaluation results of Tianjin.

Date	Predicted Value						Actual Value					
	I	II	III	IV	V	Level	I	II	III	IV	V	Level
2018/9/1	0.3742	0.4024	0	0	0	II	0.3873	0.3000	0	0	0	I
2018/9/2	0.3429	0.2484	0	0	0	I	0.3369	0.2500	0	0	0	I
2018/9/3	0.3899	0.3899	0	0	0	I	0.3286	0.0833	0	0	0	I
2018/9/4	0.4165	0.4944	0	0	0	II	0.4933	0.1167	0	0	0	I
2018/9/5	0.4325	0.4325	0	0	0	I	0.4160	0.4160	0	0	0	I
2018/9/6	0.3855	0.0981	0	0	0	I	0.3448	0.1900	0	0	0	I
2018/9/7	0.4138	0	0	0	0	I	0.4372	0	0	0	0	I
2018/9/8	0.3991	0	0	0	0	I	0.3970	0	0	0	0	I
2018/9/9	0.4137	0.3976	0	0	0	I	0.3833	0.4446	0	0	0	II
2018/9/10	0.2117	0.4074	0	0	0	II	0.2050	0.4109	0.1273	0	0	II
2018/9/11	0.2155	0.4149	0.1949	0	0	II	0.2090	0.4366	0.2727	0	0	II
2018/9/12	0.2329	0.3903	0.2889	0	0	II	0.2423	0.3796	0.1091	0	0	II
2018/9/13	0.2748	0.3510	0.3495	0	0	II	0.2624	0.3427	0.3091	0	0	II
2018/9/14	0.3118	0.3118	0	0	0	I	0.2174	0.3261	0.0727	0	0	II
2018/9/15	0.3053	0.0750	0	0	0	I	0.3111	0.0800	0	0	0	I
2018/9/16	0.2726	0.1214	0	0	0	I	0.3342	0.0750	0	0	0	I
2018/9/17	0.2436	0.2089	0	0	0	I	0.2807	0.2512	0	0	0	I
2018/9/18	0.3321	0.3321	0	0	0	I	0.2924	0.2250	0	0	0	I
2018/9/19	0.3263	0.4014	0	0	0	II	0.1878	0.4159	0	0	0	II
2018/9/20	0.3211	0.2638	0	0	0	I	0.3070	0.2000	0	0	0	I

**Table 10:** Air quality evaluation results of Shijiazhuang.

Date	Predicted Value						Actual Value					
	I	II	III	IV	V	Level	I	II	III	IV	V	Level
2018/9/1	0.3760	0.3359	0	0	0	I	0.4052	0.4000	0	0	0	I
2018/9/2	0.2113	0.4302	0	0	0	II	0.2326	0.4248	0.2000	0	0	II
2018/9/3	0.3602	0.1608	0	0	0	I	0.3749	0.2000	0	0	0	I
2018/9/4	0.3898	0.0818	0	0	0	I	0.4083	0.0500	0	0	0	I
2018/9/5	0.3464	0.0377	0	0	0	I	0.3453	0.1833	0	0	0	I
2018/9/6	0.3377	0.1469	0	0	0	I	0.3175	0.2300	0	0	0	I
2018/9/7	0.3980	0	0	0	0	I	0.4072	0	0	0	0	I
2018/9/8	0.4098	0.2360	0	0	0	I	0.3883	0.2333	0	0	0	I
2018/9/9	0.2982	0.3537	0	0	0	II	0.2500	0.3614	0	0	0	II
2018/9/10	0.2602	0.3283	0	0	0	II	0.2654	0.3243	0.0364	0	0	II
2018/9/11	0.2729	0.3297	0	0	0	II	0.2821	0.3100	0	0	0	II
2018/9/12	0.3150	0.3306	0	0	0	II	0.2610	0.3448	0	0	0	II
2018/9/13	0.2704	0.2704	0	0	0	I	0.2543	0.2400	0	0	0	I
2018/9/14	0.2224	0.3135	0	0	0	II	0.1821	0.3227	0.1750	0	0	II
2018/9/15	0.2477	0.3391	0	0	0	II	0.3245	0.3619	0	0	0	II
2018/9/16	0.2830	0.2830	0	0	0	I	0.2842	0.2842	0	0	0	I
2018/9/17	0.2858	0.2087	0	0	0	I	0.2998	0.2998	0	0	0	I
2018/9/18	0.3047	0.2141	0	0	0	I	0.2622	0.2168	0	0	0	I
2018/9/19	0.3170	0.2195	0	0	0	I	0.3291	0.2131	0	0	0	I
2018/9/20	0.2597	0.2129	0	0	0	I	0.2451	0.2197	0	0	0	I

**Table 11:** Air pollution early-warning handbook.

Level	Category	Color	Condition	Measure
I	excellent	green	satisfactory air quality	Outdoor activities are suitable for all people.
II	good	blue	acceptable air quality	The very few abnormally sensitive people should reduce outdoor activities.
III	moderate	yellow	mild pollution is unhealthy to sensitive people	Sensitive people including children, the elderly and patients with respiratory tract, cardiovascular and cerebrovascular diseases should reduce outdoor activities. Public transportation is recommended for travel.
IV	poor	red	moderate pollution is unhealthy to all people	Sensitive people should avoid outdoor activities which also need to be reduced by general people. Prefer public transportation and reduce construction and traffic dust.
V	hazardous	purple	heavy pollution is hazardous to all people	Besides above measures, road flushing and cleaning, suspension of large-scale open-air activities, outdoor personnel wear masks are all needed.
